# B Cell Kinetics upon Therapy Commencement for Active Extrarenal Systemic Lupus Erythematosus in Relation to Development of Renal Flares: Results from Three Phase III Clinical Trials of Belimumab

**DOI:** 10.3390/ijms232213941

**Published:** 2022-11-11

**Authors:** Ioannis Parodis, Alvaro Gomez, Julius Lindblom, Jun Weng Chow, Christopher Sjöwall, Savino Sciascia, Mariele Gatto

**Affiliations:** 1 Division of Rheumatology, Department of Medicine Solna, Karolinska Institutet and Karolinska University Hospital, 17176 Stockholm, Sweden; 2 Department of Rheumatology, Faculty of Medicine and Health, Örebro University, 70281 Örebro, Sweden; 3 Division of Inflammation and Infection, Department of Biomedical and Clinical Sciences, Linköping University, 58183 Linköping, Sweden; 4 Center of Research of Immunopathology and Rare Diseases and Nephrology and Dialysis, Department of Clinical and Biological Sciences, University of Turin, 10124 Turin, Italy; 5 Unit of Rheumatology, Department of Medicine, University of Padua, 35040 Padua, Italy

**Keywords:** systemic lupus erythematosus, biomarkers, renal flares, B cells, plasma cells, B lymphocyte, belimumab, biologics

## Abstract

Renal flares constitute major determinants of poor prognosis in people living with systemic lupus erythematosus (SLE). The aim of the present study was to investigate changes in B cell subsets in relation to renal flares upon initiation of standard therapy (ST) plus belimumab or placebo in patients with SLE. Using data from the BLISS-76, BLISS-SC, and BLISS Northeast Asia trials, we investigated associations of relative to baseline rapid (through week 8) and early (through week 24) percentage changes in circulating CD19^+^ B cell subsets characterised through flow cytometry, anti-dsDNA antibodies, and complement levels with the occurrence of renal flares over one year. Patients who developed renal flares showed more prominent rapid decreases in CD19^+^CD20^+^CD138^+^ short-lived plasma cells (−50.4% vs. −16.7%; *p* = 0.019) and CD19^+^CD20^-^CD27^bright^ plasmablasts (−50.0% vs. −29.9%; *p* = 0.020) compared to non-flaring patients, followed by a subsequent return. Less prominent rapid reductions in CD19^+^CD27^-^CD24^bright^CD38^bright^ transitional B cells (−42.9% vs. −75.0%; *p* = 0.038) and CD19^+^CD20^-^CD138^+^ peripheral long-lived plasma cells (−11.3% vs. −29.2%; *p* = 0.019) were seen in belimumab-treated—but not placebo-treated—patients who developed renal flares compared to belimumab-treated patients who did not. Rapid and early changes in anti-dsDNA or complement levels showed no clear association with renal flares. In summary, a rapid drop followed by a subsequent return in circulating short-lived plasma cells and plasmablasts upon treatment for active extra-renal SLE portended renal flares, indicating a need for therapeutic adjustments in patients showing such B cell patterns. Rapid decreases in transitional B cells and peripheral long-lived plasma cells upon belimumab therapy commencement may signify a greater protection against renal flares. B cell kinetics may prove useful in early drug evaluation.

## 1. Introduction

Renal flares constitute major determinants of poor prognosis in patients with systemic lupus erythematosus (SLE) since they contribute to renal and overall organ damage accrual as well as to increased disease- and treatment-related morbidity and costs [1,2,3,4]. Renal flares are coupled with increases in proteinuria and/or serum creatinine levels as well as substantial nephron loss, eventually resulting in irreversible worsening of renal function [5,6]. Risk factors for renal flares in SLE include persistently active extrarenal disease, low complement levels, and positive anti-U1RNP and anti-dsDNA antibodies [7,8], yet patient monitoring is mainly based on fluctuations in proteinuria and serum creatinine, abnormalities in the urinary sediment, and changes in serological markers, which are often subject to inconsistencies owing to different assays and timings of sample collection [9,10,11].

Belimumab blocks the soluble counterpart of B cell activating factor (BAFF; also known as B lymphocyte stimulator, BLyS) and has been used for the treatment of SLE for over a decade [12]. It has shown ability to induce durable disease control and reduce the risk of disease flares, including renal flares, in clinical trials and several real-life observational studies [13,14,15,16,17,18], and after a recent phase III lupus nephritis (LN)-specific clinical trial [19], belimumab received approval from regulatory agencies for use as an add-on therapy in addition to standard immunosuppressive therapy (mycophenolate mofetil or low-dose intravenous cyclophosphamide) in patients with SLE and active renal involvement [20]. Still, some patients may develop renal flares during belimumab therapy, including de novo LN, as exemplified in a recent report [21], mandating identification of patient profiles with susceptibility to develop renal flares despite immunosuppressive therapy, including therapy with belimumab, as an urgent need towards determination of individualised therapeutic modifications required to prevent renal flares in the short term and kidney function loss over the longer term.

In this regard, identification of reproducible biological changes occurring soon after treatment initiation that are associated with renal flares could introduce a novel concept in surveillance upon commencement of a new therapy, early evaluation of its effectiveness, and evaluation of the need for treatment modification in selected patients. Hence, in this study, we aimed at investigating early changes in B cell and plasma cell subsets in relation to the development of renal flares during non-biological standard therapy (ST) plus belimumab or placebo within the frame of three phase III clinical trials of belimumab in SLE.

## 2. Results

### 2.1. Patient Characteristics

Demographics and clinical and serological data of the patients, including comparisons between patients who developed and patients who did not develop renal flares through week 52, are reported in Table 1. Patients who developed renal flares were younger at baseline (34.6 ± 11.6 years vs. 39.5 ± 11.9 years; *p* = 0.001). Higher proportions of patients among those who developed renal flares were on glucocorticoids at baseline (93.8% vs. 81.5%; *p* = 0.012) and were of Asian ancestry (42.2% vs. 14.7%; *p* < 0.001) compared to patients who did not develop renal flares, while lower proportions of patients among those who developed renal flares were White/Caucasian (35.9% vs. 63.5%; *p* < 0.001). A total of 633/1715 patients (36.9%) had a history of or current renal SLE at baseline (renal BILAG A–D), and 152 patients (8.9%) had active renal disease (renal BILAG A or B). A higher proportion of patients with renal BILAG A–B developed renal flares through week 52 compared to patients who did not (28.1% vs. 8.1%; *p* < 0.001). Detailed information about BILAG-based organ involvement at baseline is presented in Supplementary Table S1.

The corresponding results from comparisons between patients who developed renal flares through the end of follow-up—i.e., week 52 in BLISS-SC and BLISS-NEA but week 76 in BLISS-76—are presented in Supplementary Table S2. Table 2 shows baseline B cell and plasma cell counts as well as comparisons between patients who developed renal flares through week 52 and patients who did not. In Table 2, results are stratified by study to account for batch variations in cell analyses across studies, and the corresponding results for renal flares through the end of follow-up are detailed in Supplementary Table S3.

### 2.2. Associations with Renal Flares Occurring during Follow-Up

In the pooled datasets, 64/1715 patients (3.7%) developed at least one renal flare through week 52, and 69/1715 patients (4.0%) developed at least one renal flare through the end of the study period, i.e., including the follow-up period of week 52–76 in BLISS-76. Among patients who developed renal flares, the first renal flare through week 52 occurred after a mean time of 160.9 ± 102.9 days from baseline, and the first renal flare throughout the entire follow-up was documented after a mean time of 181.7 ± 124.5 days from baseline.

### 2.3. B Cell Changes

In the entire cohort (all treatment arms), patients who developed at least one renal flare through week 52 showed a more profound rapid decrease in CD19^+^CD20^+^CD138^+^ short-lived plasma cells (−50.4% vs. −16.7%; *p* = 0.019) and CD19^+^CD20^-^CD27^bright^ plasmablasts (−50.0% vs. −29.9%; *p* = 0.020) compared with patients who did not develop renal flares in logistic regression analysis after adjustment for potential confounders, as described in the Methods. In patients who developed renal flares, this initial drop in the aforementioned cell subsets was followed by a subsequent increase, while in patients who did not develop renal flares, these lymphocyte subsets continued the declining trend, as detailed in Supplementary Tables S4–S6. In contrast, patients who flared showed less prominent CD19^+^CD20^-^CD138^+^ peripheral long-lived plasma cells through week 24 compared to patients who did not (−10.4% vs. −38.8%; *p* = 0.028).

**Figure 1:**
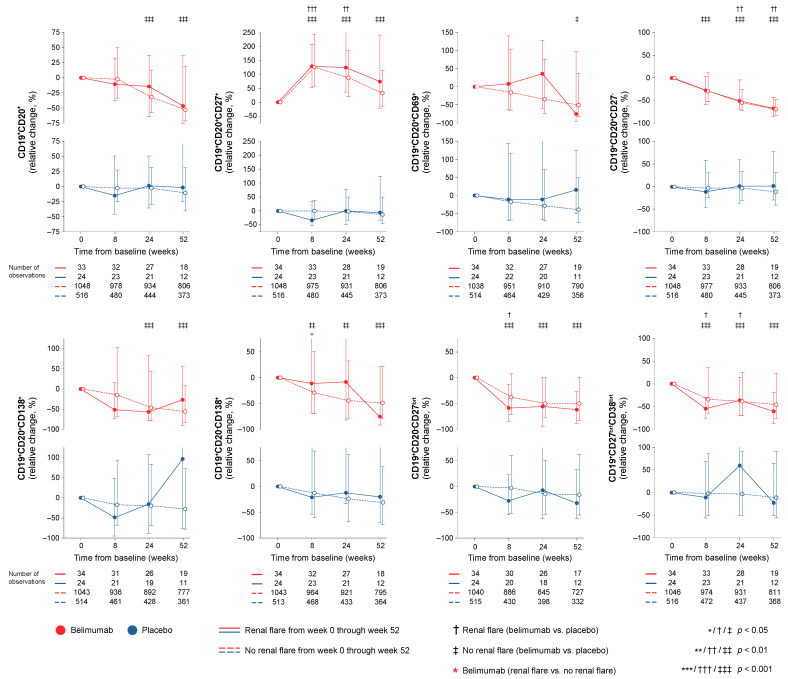
B cell alterations in relation to renal flares. The graphs delineate relative to baseline percentage changes in selected B cell and peripheral plasma cell subsets in patients who developed at least one renal flare during the study period (continuous lines) and patients who did not (dashed lines). Comparisons between patients who flared and patients who did not were conducted using multivariable logistic regression analysis to account for potential confounders and are illustrated for patients who received non-biological standard therapy plus belimumab (red lines) and patients who received non-biological standard therapy alone (blue lines). Comparisons between treatment arms were conducted using non-parametrical Mann–Whitney *U* tests. Whiskers indicate the interquartile range of distributions. The number of patients with available data at each time point is indicated for each patient subgroup.

Among patients who received add-on belimumab, patients who developed at least one renal flare showed a less profound rapid decrease from baseline through week 8 in CD19^+^CD20^-^CD138^+^ peripheral long-lived plasma cells (−11.3% vs. −29.2%; *p* = 0.019) compared to patients who did not develop renal flares (Figure 1). Among patients who received standard therapy alone, no differences were seen in rapid or early changes in B cell or plasma cell subsets between patients who developed renal flares through week 52 and patients who did not.

Results from analysis in the entire cohort and analysis stratified by treatment arm for renal flares throughout the entire follow-up (baseline through week 52 in BLISS-SC and BLISS-NEA and through week 76 in BLISS-76) are detailed in Supplementary Tables S7–S9. Supplementary Tables S4–S9 also detail comparisons of changes in B cell and plasma cell subsets between patients who received ST plus belimumab and patients who received ST plus placebo.

**Figure 2:**
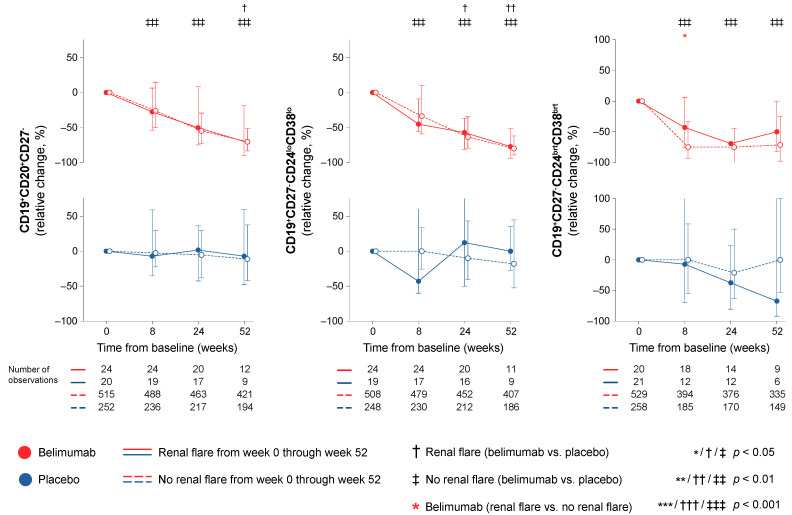
Transitional and naïve B cell alterations in relation to renal flares. The graphs delineate relative to baseline percentage changes in transitional and naïve B cell subsets in patients who developed at least one renal flare during the study period (continuous lines) and patients who did not (dashed lines) in a subanalysis of data from the BLISS-SC trial. Comparisons between patients who flared and patients who did not were conducted for patients with available data, stratified into patients who received non-biological standard therapy plus belimumab (red lines) and patients who received non-biological standard therapy alone (blue lines). P values are derived from non-parametric Mann–Whitney *U* tests. Whiskers indicate the interquartile range of distributions. The number of patients with available data at each time point is indicated for each patient subgroup.

In a subgroup analysis of the CD19^+^CD20^+^CD27^-^ B cell subset in the BLISS-SC trial, a less prominent rapid decrease in CD19^+^CD27^-^CD24^bright^CD38^bright^ transitional B cells was seen in belimumab-treated patients who developed at least one renal flare through week 52 compared with belimumab-treated patients who did not (−42.9% vs. −75.0%; *p* = 0.038), as illustrated in Figure 2. In contrast, no differences were seen regarding rapid or early changes in transitional B cells between patients who developed renal flares and patients who did not among patients who were exposed to non-biological ST alone (Figure 2). Moreover, no differences were observed regarding changes in CD19^+^CD27^-^CD24^low^CD38^low^ naïve B cells between flaring and non-flaring patients. Detailed results from this analysis are presented in Supplementary Table S10.

### 2.4. Serological Markers

**Figure 3:**
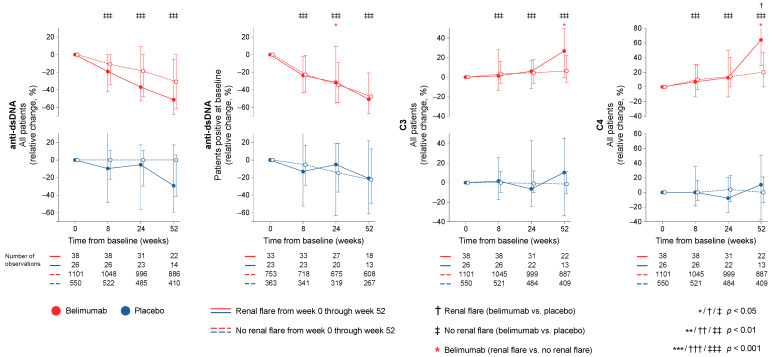
Changes in serological markers in relation to renal flares. The graphs delineate relative to baseline percentage changes in anti-dsDNA, C3, and C4 levels in patients who developed at least one renal flare during the study period (continuous lines) and patients who did not (dashed lines). Comparisons between patients who flared and patients who did not were conducted using multivariable logistic regression analysis to account for potential confounders and are illustrated for patients who received non-biological standard therapy plus belimumab (red lines) and patients who received non-biological standard therapy alone (blue lines). For anti-dsDNA levels, a separate analysis for patients with positive anti-dsDNA levels (≥30 IU/mL) at baseline is also demonstrated. Comparisons between treatment arms were conducted using non-parametrical Mann–Whitney *U* tests. Whiskers indicate the interquartile range of distributions. The number of patients with available data at each time point is indicated for each patient subgroup. Anti-dsDNA: anti-double-stranded DNA antibodies; C3: complement component 3; C4: complement component 4.

In the entire cohort (all treatment arms) patients who developed at least one renal flare through week 52 had higher baseline anti-dsDNA levels (median; IQR: 256.0; 97.5–632.0 IU/mL vs. 90.0; 29.0–275.3 IU/mL; *p* < 0.001) and lower C3 (median; IQR: 75.0; 57.3–91.5 mg/dL vs. 96.0; 75.0–119.0 mg/dL; *p* < 0.001) and C4 levels (median; IQR: 11.0; 7.0–16.0 mg/dL vs. 15.0; 9.0–22.0 mg/dL; *p* < 0.001) compared to patients who did not develop renal flares. Rapid and early changes of anti-dsDNA antibody levels, C3 levels, and C4 levels did not differ between patients who developed renal flares through week 52 and patients who did not. Similar patterns were seen in analysis stratified by treatment arms (Figure 3). The results are detailed in Supplementary Tables S4–S6, and the corresponding results for renal flares through week 76 are detailed in Supplementary Tables S7–S9. These tables also detail comparisons of changes in serological markers between patients who received ST plus belimumab and patients who received ST plus placebo.

### 2.5. Analyses in Relation to the First Documented Renal Flare

To further understand the observed kinetics of B cell and plasma cell subsets, anti-dsDNA antibody levels, and complement levels, we investigated absolute cell counts and anti-dsDNA, C3, and C4 levels in relation to the time of the first documented renal flare. More specifically, we compared the distributions of absolute cell counts and anti-dsDNA, C3, and C4 levels measured at the most adjacent timepoint before (median time: −7.9; IQR: −15.4–−4.1 weeks) and after (median time: 10.9; IQR: 3.9–12.7 weeks) the first renal flare. In this analysis, absolute CD19^+^CD20^+^CD138^+^ short-lived plasma cell counts displayed a decrease between the last available measurement prior to the first documented renal flare (median: 422.5 cells/mL; IQR: 274.5–567.9 cells/mL) and the first available measurement after the renal fare (median: 183.0 cells/mL; IQR: 130.4–301.0 cells/mL; *p* = 0.035). In contrast, C4 levels displayed an increase from the first available measurement prior to renal flare (median: 9.5 mg/dL; IQR: 7.0–13.8 mg/dL) to the first measurement after the renal flare (median: 12.5 mg/dL; IQR 7.3–18.0 mg/dL; *p* = 0.011). All other cell subsets, anti-dsDNA antibody levels, and C3 levels showed no statistically significant change before and after the first documented renal flare (*p* > 0.05 for all comparisons).

## 3. Discussion

In this study, we investigated alterations across different circulating B cell subsets upon treatment for active SLE and their association with occurrence of renal flares. We showed that a course in short-lived plasma cells and plasmablasts characterised by a rapid decrease followed by a subsequent return was associated with the occurrence of renal flares; this pattern may thus signify a need for treatment modifications in selected patients. While this drop–return pattern was seen both in patients treated with add-on belimumab and patients treated with non-biological ST alone, the drop in plasmablasts was more prominent in belimumab-treated patients irrespective of the development of renal flares. It is worth noting that belimumab was herein shown to induce declining trends in plasma cell subsets early on upon treatment initiation, while in previous research, data on the effect of belimumab on plasma cells have been conflicting [22,23,24]. This may be due to the large study population in the present work and the resulting power amplification in statistical calculations as well as the detailed characterisation of peripheral plasma cells into different subsets. A similar pattern of an initial drop and subsequent return in memory B cells discriminated patients on non-biological ST who flared from those who did not flare in unadjusted analysis, which, however, did not reach statistical significance after adjustment for confounders. In contrast, circulating memory B cells showed a rapid increase upon belimumab treatment regardless of the occurrence of renal flares. This increase in circulating memory B cells occurring short time after commencement of belimumab therapy has been described in previous research [22,23,24] and has been speculated to be related to a secondary defect in their trafficking receptors [25]. Albeit not unexpected in light of its mode of action, belimumab was shown to induce a rapid and sustained decline in transitional B cells, which was less prominent in belimumab-treated patients who developed renal flares. This finding is novel and suggests that transitional B cell kinetics may be an early indicator of successful treatment with belimumab, with pronounced rapid reductions signifying a better protection against renal flares. Lastly, while high levels of anti-dsDNA antibodies and low levels of C3 and C4 at baseline were associated with renal flare development, rapid or early changes in these traditional serological markers were not.

While renal flares constitute a major contributing factor of poor long-term prognosis in patients with SLE [26,27,28], traditional biomarkers do not satisfactorily predict their occurrence [10], especially when evaluating the likelihood of renal flare-up upon commencing therapy for active SLE. In conformity with the above, early changes in anti-dsDNA or complement levels did not discriminate between patients who developed renal flares and patients who did not in the present study. Moreover, while several studies have shown that attainment of low proteinuria levels at one year of therapy for lupus nephritis is coupled with a better long-term renal prognosis, failure to attain this target was not clearly predictive of poor outcome [27,29,30], and another study found no clear association between proteinuria levels at one year of therapy and subsequent renal flares [31]. In contrast, this latter study revealed that active glomerular inflammatory lesions in per-protocol repeat kidney biopsies after the initial phase of therapy were predictive of subsequent renal flares [31]. Although direct examination of the kidney biopsy is considered the gold standard for determination of therapeutic need, its invasiveness makes it inappropriate in several cases, especially when the purpose is to predict future events rather than confirmation of activity and justification of therapy upon clinical indications. Moreover, while several theories exist, the exact mechanisms underlying inflammatory kidney injury in patients with SLE are not fully elucidated, and it is still unclear whether immune activation preceding nephritis starts in the periphery or in situ [5].

Various functions of B cells have been implicated in the pathogenesis of LN, including the production of inflammatory mediators or potentially nephritogenic autoantibodies and cytotoxicity mediated by interactions with components of the complement system. Studies of murine lupus have shown that B cells infiltrating the kidney tissue secrete antibodies with various antigen specificities and contribute to in situ immune complex formation [32,33,34]. Also in human studies, germinal-centre-like structures and T and B cell aggregates in the kidney have been shown to promote in situ secretion of pathogenic antibodies and immune complexes [35,36]. Moreover, in response to evidence that B cell depletion prevents or delays the onset of glomerulonephritis in lupus-prone mice [37,38] and induces complete or partial clinical remission in patients with lupus nephritis [39,40,41], B cell modulation with the BAFF inhibiting monoclonal antibody belimumab was recently shown successful in a lupus-nephritis-specific phase III clinical trial [19] and received approval for the treatment of this lupus manifestation. Moreover, the B-cell-depleting agent obinutuzumab has entered a phase III protocol after promising results in a phase II trial [41]. It is, however, worth noting that where the ability of B cells to secrete antibodies is impeded, lupus-prone mice have also been shown to develop nephritis [42], implying that B cell functions other than antibody production, such as antigen presentation or cytokine production, may also contribute to inflammatory kidney injury. Altogether, investigation of biological events in the periphery that can be anticipated to reflect the inflammatory activity in the kidney preceding renal flares has merit, and we hypothesised that kinetics of peripheral B cell and plasma cell subsets might prove to be a useful surveillance tool in this regard.

A kinetics pattern of a rapid drop in short-lived plasma cells and plasmablasts with a subsequent return was associated with the development of renal flares, while patients who did not develop renal flares showed more gradual decreases. Interestingly, this drop–return pattern was prominent in patients who received non-biological ST alone while the returning trend in these cell subsets was less pronounced or absent among patients who received add-on belimumab. It is worth noting that belimumab was herein shown to induce declining trends in certain plasma cell subsets early on upon treatment initiation, in part contrasting previous conflicting data [22,23,24], potentially owing to the large study population and resulting power amplification in statistical calculations as well as the detailed characterisation of peripheral plasma cells into different subsets. Another point of striking interest was the rapid decrease in peripheral long-lived plasma cells in belimumab-treated patients who did not develop renal flares, which was more prominent than in belimumab-treated patients who flared. In contrast, no such discriminative ability was observed for long-lived plasma cell kinetics in patients who were on non-biological ST alone. While the origin of long-lived plasma cells found in the periphery is unclear, this finding is in an intuitive direction and may prove useful in the early evaluation of belimumab therapy, signifying a better response and thus a protection against renal flare development in patients showing rapid reductions.

A similar pattern of an initial drop and subsequent return in memory B cells was seen in patients on non-biological ST who developed renal flares, while circulating memory B cells showed a rapid increase upon belimumab treatment regardless of renal flare occurrence. This increase in circulating memory B cells seen short time after initiation of belimumab treatment has been described in previous research [22,23,24], and it has been speculated to be related to a secondary defect in their trafficking receptors [25]. Thus, it may be argued that gradual decreases in selected B cell subsets may signify a durable response to treatment in terms of protection against renal flares, while return trends may be indicative of a rebound B cell enrichment or relative enrichment of certain subsets within the B cell pool.

While the difference did not reach statistical significance after adjustments, changes in activated B cells displayed numerically prominent differential patterns within the belimumab-treated population, with patients who developed renal flares showing increasing trends and patients who did not flare showing declines. The separation trend was seen at week 8, yielding an absolute difference of 23.9%, but was more prominent in the comparison of week 24, yielding an absolute difference of 70.7%. Despite the lack of statistical significance, the direction of this observation is intuitive, with B cells carrying activation markers accumulating towards a renal flare and decreasing activated B cells signifying favourable response to belimumab and protection against renal flares, warranting further study of this B cell subset in relation to responses to belimumab therapy.

In a subgroup analysis aiming at a better characterisation of the naïve and transitional B cells, transitional B cells showed rapid and sustained reductions in belimumab-treated patients, which were more prominent in patients who did not develop renal flares. In contrast, patients treated with non-biological ST alone showed less prominent decreases in transitional B cells, which did not distinguish flaring from non-flaring patients. Based on its mode of action, belimumab is expected to impact transitional and naïve B cells [43,44], while the more prominent decline in transitional B cells in belimumab-treated patients who were protected against renal flares may be speculated to be due to an augmented BAFF effect on transitional B cells in an environment of declining numbers of naïve B cells. Supportive of the latter may also be considered the previously documented increase in BAFF levels upon exposure to belimumab therapy [17].

While high levels of anti-dsDNA antibodies and low levels of C3 and C4 at baseline signified patients at risk for renal flares during follow-up, their rapid and early kinetics in response to therapy were not found to have any predictive value. Lastly, the higher levels of C4 and lower levels of short-lived plasma cells measured after the first documented renal flare compared with the last available measurement prior to the flare may be due to an effect of the glucocorticoid and/or immunosuppressive rescue therapy given to treat the observed flare. It is important to underline that our findings are rather hypothesis-generating and not intended to suggest the substitution of traditional serological markers with B cell and peripheral plasma cell kinetics. They are rather intended to suggest the use of both in a complemental fashion while monitoring drug efficacy, especially belimumab or other B cell targeting therapies, to obtain a better insight into the biological drug response and facilitate early treatment evaluation based on evidence-based expectations for subsequent clinical outcomes.

It is important to acknowledge that this study included a selected SLE population mainly displaying musculoskeletal and mucocutaneous activity at baseline; in total, 36.9% of the study participants had current or past renal involvement at baseline. Together with the short follow-up time, this explains the overall low incidence of renal flares during the study period and limits the generalisability of the findings in real-world SLE populations of higher percentages of renal involvement [1,5]. On the other hand, the study encompassed a large number of patients that commenced therapy for active, autoantibody-positive extrarenal disease, who are expected to be at a certain risk for developing renal flares [7] and were followed up in a structured manner which allowed for the detection of patterns of lymphocyte alterations over time after treatment commencement.

While SLE populations more enriched in active renal disease might yield a higher renal flare rate, it is of clinical relevance to also investigate renal flare development in a population commencing therapy for active extrarenal disease for several reasons. Firstly, because treatment given for extrarenal disease is not necessarily protective against development of renal activity and understanding how to prevent this is warranted. Secondly, a proportion of the study participants had active renal SLE (8.9%), and many had a history of renal involvement, albeit quiescent (5.7%), or stable (22.3%) renal disease at baseline. Thirdly, cases of de novo lupus nephritis development after commencement of belimumab therapy in patients with no prior renal SLE have been reported [21,45]. Hence, especially in light of the recent approval of add-on belimumab for active lupus nephritis [19], it is important to understand which patient subgroups are protected against renal flares during belimumab therapy and which patients are not. In the present investigation, the proportion of individuals who developed renal flares differed from those who did not in favour of belimumab only within patients treated with the low dose of i.v. belimumab (1 mg/kg). While the approved dose of add-on i.v. belimumab is 10 mg/kg, this dose was tested in patients with active LN and high levels of proteinuria, resulting in an increased drug clearance [19]. Altogether, the dose of belimumab in SLE patients with low-grade or no proteinuria may still require investigation in relation to drug effects on B cells with regulatory properties, as previously postulated [21], and indirectly supported by the prominent reductions of IL-10 upon belimumab therapy commencement for active extrarenal SLE [46].

## 4. Materials and Methods

### 4.1. Study Population

We analysed prospectively collected longitudinal data from patients with active SLE who participated in three multicentre, randomised, double-blind, placebo-controlled trials comparing belimumab (administered intravenously or subcutaneously) with placebo—i.e., BLISS-76 (NCT00410384; *n* = 819) [47], BLISS-SC (NCT01484496; *n* = 836) [48], and BLISS Northeast Asia (NEA; NCT01345253; *n* = 60) [49]—in a post hoc manner. The study population (*n* = 1715) was selected based on the a priori flow cytometry analysis plan for each one of the BLISS trials and therefore based on the availability of data on B cell subset counts, selected serological markers, and clinical data needed to determine renal flares. In the BLISS programmes, belimumab or placebo was administered on top of ST, including antimalarial agents, glucocorticoids, immunosuppressive agents, or combinations thereof.

In terms of design, the three trials were similar. Briefly, all patients were required to have a Safety of Estrogens in Lupus Erythematosus National Assessment-Systemic Lupus Erythematosus Disease Activity Index (SELENA-SLEDAI) [50] score ≥6 (BLISS-76) or ≥8 (BLISS-SC and BLISS-NEA) and had to be autoantibody-positive (antinuclear antibody titres ≥1:80 and/or anti-double stranded (ds)DNA levels ≥30 IU/mL) at the screening visit. All patients had received stable dosages of ST for at least 30 days prior to baseline. For BLISS-76 and BLISS-NEA, belimumab or placebo were administered intravenously on days 0, 14, and 28, and every 4th week thereafter through week 48 (NEA) or week 72 (BLISS-76). In BLISS-SC, belimumab 200 mg or placebo was administered subcutaneously weekly through week 52, on top of non-biological ST. Progressive restrictions were imposed during the trial periods on concurrent immunosuppressive and antimalarial medications, as well as glucocorticoid intake. The primary endpoint in all trials was the proportion of responders at week 52, with response being determined using the composite SLE Responder Index (SRI)-4 [51]. The similar trial design and endpoints allowed pooling of the data to increase power during statistical analyses.

### 4.2. Clinical Definitions

Renal flare was defined as the occurrence of one or more of the following features on two or more consecutive visits during the study period: (i) a reproducible increase in 24 h urine protein equivalent levels to >1 g if the baseline value was <0.2 g, >2 g if the baseline value was 0.2 g to 1 g, or >2 times the baseline value if the baseline value was >1 g; (ii) a reproducible increase in serum creatinine by ≥20% or ≥0.3 mg/dL, accompanied by proteinuria (equivalent to >1 g/24 h), haematuria (≥4 red blood cells per high power field) and/or red blood cell casts; (iii) treatment-emergent reproducible haematuria (≥11 to 20 red blood cells per high power field) or a reproducible increase in haematuria by 2 grades compared to baseline associated with 25% dysmorphic red blood cells, glomerular in origin, exclusive of menses, and accompanied by either an ≥0.8 g increase in 24 h proteinuria (or equivalent amount measured by other means, such as the urinary protein to creatinine ratio) or new red blood cell casts [18]. Occurrence of renal flare was determined every fourth week during the study period.

History of or current renal involvement was defined as a renal score of A–D in the classic British Isles Lupus Assessment Group Index (BILAG) [52], while no history of renal involvement was defined a renal classic BILAG E. Active renal SLE was defined as a renal classic BILAG A or B. Organ damage was determined with the Systemic Lupus International Collaborating Clinics (SLICC)/American College of Rheumatology (ACR) Damage Index (SDI) [53].

### 4.3. Determination of B Cell Subsets and Serological Markers

Peripheral B cell and plasma cell subsets were determined via flow cytometry, and the gating strategy for cell separation was employed within the frame of the BLISS study programmes [47,48,49]. Flow cytometry was performed on samples captured at weeks 8, 24, and 52 in BLISS-76 and BLISS-SC, and at weeks 8, 12, and 52 in BLISS-NEA. The cell subsets were classified into total peripheral CD19^+^CD20^+^ B cells, CD19^+^CD20^+^CD69^+^ activated B cells, CD19^+^CD20^+^CD27^-^ naïve B cells, CD19^+^CD20^+^CD27^+^ memory B cells, CD19^+^CD20^-^CD27^bright^ plasmablasts, CD19^+^CD20^+^CD138^+^ short-lived plasma cells, CD19^+^CD20^-^CD138^+^ long-lived plasma cells, and CD19^+^CD38^bright^CD27^bright^ SLE-associated plasma cells based on previous works deriving from the BLISS trials and other literature [23,54,55,56]. In a subgroup analysis to better characterise the CD19^+^CD20^+^CD27^-^ cell subset performed in the population from the BLISS-SC trial, which encompassed a more detailed gating strategy, CD19^+^CD27^-^CD24^bright^CD38^bright^ designated transitional B cells, and CD19^+^CD27^-^CD24^low^CD38^low^ designated a befitting naïve B cell subset [57]. Levels of anti-dsDNA, C3, and C4 were determined within the frame of the BLISS programmes [47,48,49] and were made available through the Clinical Study Data Request (CSDR) consortium.

We analysed changes in B cell subsets and serum levels of anti-dsDNA, C3, and C4 that occurred through weeks 8, 24, and 52 relative to baseline (i.e., treatment initiation). The changes in B cell subsets between baseline and week 12 in the 60 patients from the BLISS-NEA trial were pooled with changes in B cell subsets between baseline and week 24 in the rest of the study population and were thus collectively termed changes through week 24. Changes occurring through week 8 were deemed rapid, and changes occurring through week 24 were deemed early; further changes were referred to as delayed. We next investigated associations between changes in B cell or plasma cell subsets or changes in serological markers and renal flares occurring until week 52 in a first analysis; throughout the entire follow-up, i.e., through week 52, for BLISS-SC and BLISS-NEA; and through week 76 for BLISS-76 in a subsequent analysis.

### 4.4. Ethics

Data from the BLISS trials were made available by GlaxoSmithKline (Uxbridge, UK) through the CSDR consortium. The trial protocols were approved by regional ethics review boards for all participating centres and complied with the ethical principles of the Declaration of Helsinki. Written informed consent was obtained from all study participants prior to enrolment. The present study was approved by the Swedish Ethical Review Authority (reference: 2019-05498).

### 4.5. Statistical Analysis

Descriptive statistics are reported as means and standard deviations or medians and interquartile ranges for continuous variables, while frequencies and percentages are reported for categorical variables. For comparisons of patient characteristics between patients who developed renal flares and patients who did not, non-parametrical Mann–Whitney *U* tests were used for continues variables, and chi-squared (*χ*^2^) or Fisher’s exact tests were used for binomial variables as appropriate. Comparisons of distributions of relative to baseline changes between flaring and non-flaring patients were conducted using multivariable logistic regression models; apart from the main exposure under investigation (i.e., relative to baseline percentage changes through week 8, week 24, or week 52 in B cell subset counts or serum levels of serological markers), other covariates in the models included age, ethnicity, glucocorticoid use, and belimumab use. Adjustment for belimumab use was not applicable in models stratified by treatment arm. Results from the logistic regression analyses are presented as the coefficient, odds ratio (OR), 95% confidence interval (CI), and P value for the main exposure in the respective multivariable logistic regression model.

In subgroup analyses from the BLISS-SC for a more in-depth characterisation of naïve and transitional B cells, the corresponding comparisons were performed using non-parametrical Mann–Whitney *U* tests due to the lack of a sufficient number of events (renal flares) limiting us from performing multivariable logistic regression analysis. Taking the randomisation in the clinical trials into consideration, comparisons between treatment arms were derived from non-parametrical Mann–Whitney *U* tests. Comparisons of distributions of related (paired) samples before and after the occurrence of the first recorded renal flare were conducted using the non-parametrical Wilcoxon signed-rank test. *p* values below 0.05 were deemed statistically significant. All analyses were performed using the R version 4.01 software (R Foundation for Statistical Computing, Vienna, Austria). The GraphPad Prism software version 9 (La Jolla, CA, USA) was used for the preparation of graphs.

## 5. Conclusions

To summarise, we showed that changes in the circulating B cell compartment in patients undergoing immunosuppressive treatment for active extra-renal SLE may help identify patients at risk for impending development of a renal flare and might hence have a place in disease surveillance as a complement to traditional parameters. Our findings provide implications that B cell kinetics with ability to inform about imminent renal flares differ between patients treated with non-biological standard therapy and patients receiving add-on belimumab owing to the effects of BAFF inhibition on the B cell compartment; this renders identification of therapy-specific patterns of B cell alterations of particular importance. The most striking results from the present study suggested that prominent rapid decreases in transitional B cells and peripheral long-lived plasma cells may signify a more favourable response to belimumab therapy and protection against renal flares.

## Figures and Tables

**Table 1:** Characteristics of patients who developed renal flares vs. patients who did not from baseline through week 52 in the pooled BLISS study population.

	All Patients	Renal Flare	No Renal Flare	*p* Value	OR	95% CI (OR)	RR	95% CI (RR)
	*n* = 1715	*n* = 64	*n* = 1651					
**Patient characteristics**								
Age at baseline (years)	39.3 ± 11.9	34.6 ± 11.6	39.5 ± 11.9	**0.001**	N/A	N/A	N/A	N/A
Female sex	1608 (93.8%)	62 (96.9%)	1546 (93.6%)	0.294	2.11	0.51–8.73	2.06	0.51–8.32
Ancestry								
Asian	270 (15.7%)	27 (42.2%)	243 (14.7%)	**<0.001**	4.23	2.53–7.07	3.91	2.42–6.30
Black/African American	204 (11.9%)	8 (12.5%)	196 (11.9%)	0.879	1.10	0.52–2.35	1.10	0.53–2.27
Indigenous American *	170 (9.9%)	6 (9.4%)	164 (9.9%)	0.883	0.94	0.40–2.21	0.94	0.41–2.15
White/Caucasian	1071 (62.4%)	23 (35.9%)	1048 (63.5%)	**<0.001**	0.32	0.19–0.54	0.34	0.20–0.56
**Clinical data**								
SLE duration at baseline (years)	5.1 (1.6–10.6)	4.1 (1.1–10.0)	5.1 (1.6–10.6)	0.202	N/A	N/A	N/A	N/A
BILAG renal								
A	10 (0.6%)	2 (3.1%)	8 (0.5%)	0.051	6.63	1.38–31.85	5.50	1.55–19.46
B	142 (8.3%)	16 (25.0%)	126 (7.6%)	**<0.001**	4.03	2.23–7.31	3.69	2.15–6.33
C	383 (22.3%)	29 (45.3%)	354 (21.4%)	**<0.001**	3.04	1.83–5.04	2.88	1.79–4.65
D	98 (5.7%)	6 (9.4%)	92 (5.6%)	0.198	1.75	0.74–4.17	1.71	0.76–3.86
E	1082 (63.1%)	11 (17.2%)	1071 (64.9%)	**<0.001**	0.11	0.06–0.22	0.12	0.06–0.23
A–B	152 (8.9%)	18 (28.1%)	134 (8.1%)	**<0.001**	4.43	2.50–7.86	4.02	2.40–6.76
Treatment at baseline								
Glucocorticoids	1405 (81.9%)	60 (93.8%)	1345 (81.5%)	**0.012**	3.41	1.23–9.46	3.31	1.21–9.04
AMA ^†^	1099 (64.1%)	36 (56.3%)	1063 (64.4%)	0.183	0.71	0.43–1.18	0.72	0.44–1.17
Immunosuppressants ^‡^	882 (51.4%)	39 (60.9%)	843 (51.1%)	0.121	1.50	0.90–2.49	1.47	0.90–2.41
Azathioprine	336 (19.6%)	15 (23.4%)	321 (19.4%)	0.430	1.27	0.70–2.29	1.26	0.71–2.21
Methotrexate	248 (14.5%)	7 (10.9%)	241 (14.6%)	0.414	0.72	0.32–1.59	0.73	0.34–1.57
Mycophenolate mofetil or sodium	243 (14.2%)	12 (18.8%)	231 (14.0%)	0.284	1.42	0.75–2.70	1.39	0.76–2.56
Trial intervention								
Placebo	576 (33.6%)	26 (40.6%)	550 (33.3%)	0.224	1.37	0.82–2.28	1.35	0.83–2.21
Belimumab	1139 (66.4%)	38 (59.4%)	1101 (66.7%)	0.224	0.73	0.44–1.21	0.74	0.45–1.20
i.v. 1 mg/kg	271 (15.8%)	2 (3.1%)	269 (16.3%)	**0.005**	0.17	0.04–0.68	0.17	0.04–0.70
i.v. 10 mg/kg	312 (18.2%)	10 (15.6%)	302 (18.3%)	0.587	0.82	0.42–1.64	0.83	0.43–1.62
s.c. 200 mg	556 (32.4%)	26 (40.6%)	530 (32.1%)	0.153	1.45	0.87–2.41	1.43	0.88–2.32
**Serological markers at baseline**								
C3; mg/dL	95.0 (74.0–118.0)	75.0 (57.3–91.5)	96.0 (75.0–119.0)	**<0.001**	N/A	N/A	N/A	N/A
C4; mg/dL	15.0 (9.0–22.0)	11.0 (7.0–16.0)	15.0 (9.0–22.0)	**<0.001**	N/A	N/A	N/A	N/A
anti-dsDNA; IU/mL (all patients)	95.0 (29.0–288.0)	256.0 (97.5–632.0)	90.0 (29.0–275.3)	**<0.001**	N/A	N/A	N/A	N/A
anti-dsDNA; IU/mL (patients positive at baseline)	167.0 (89.0–497.3); *n* = 1172	279.0 (137.3–664.5); *n* = 56	163.5 (86.5–490.8); *n* = 1116	**0.003**	N/A	N/A	N/A	N/A

Data are presented as the number (percentage), mean ± standard deviation, or median (interquartile range), as appropriate. Additionally, odds ratio (OR) and the corresponding 95% confidence interval (CI), as well as risk ratio (RR) and the corresponding 95% CI, are indicated. In case of missing values, the total number of patients with available data is indicated. Percentages are derived using the total number of patients in the respective column as the denominator (i.e., all patients, patients who developed renal flares and patients who did not develop renal flares). P values were derived from non-parametrical Mann–Whitney U tests for continues variables and chi-squared (*χ*^2^) or Fisher’s exact tests for binomial variables, as appropriate. Statistically significant P values are in bold. * Alaska Native or American Indian from North, South, or Central America. ^†^ Hydroxychloroquine, chloroquine, mepacrine, mepacrine hydrochloride, or quinine sulfate. ^‡^ Azathioprine, cyclosporine, oral cyclophosphamide, leflunomide, methotrexate, mizoribine, mycophenolate mofetil, mycophenolate sodium, or thalidomide. AMA: antimalarial agents; C3: complement component 3; C4: complement component 4; CI: confidence interval; i.v.: intravenous; N/A: not applicable; OR: odds ratio; RR: risk ratio; s.c.: subcutaneous; SLE: systemic lupus erythematosus; SRI-4: SLE Responder Index 4.

**Table 2:** B cell subset counts at baseline in patients who developed renal flares vs. patients who did not from baseline through week 52 in the BLISS-76, BLISS-SC, and BLISS Northeast Asia study populations.

B Cell Subsets	All Patients	Renal Flare	No Renal Flare	*p* Value
**BLISS-76**				
	*n* = **819**	*n* = **9**	*n* = **810**	
**CD19^+^CD20^+^** (×10^3^/mL)	91.5 (43.0–176.0); *n* = 756	95.5 (25.0–123.5); *n* = 8	91.0 (43.3–178.0); *n* = 748	0.386
**CD19^+^CD20^+^CD27^+^** (×10^3^/mL)	14.0 (6.0–27.0); *n* = 756	13.5 (3.3–23.3); *n* = 8	14.0 (6.0–27.0); *n* = 748	0.456
**CD19^+^CD20^+^CD69^+^** (/mL)	2096.5 (938.3–4350.8); *n* = 744	2769.5 (708.3–9099.3); *n* = 8	2096.5 (938.3–4327.0); *n* = 736	0.531
**CD19^+^CD20^+^CD27^-^** (×10^3^/mL)	75.0 (33.0–143.0); *n* = 756	77.5 (20.0–103.0); *n* = 8	75.0 (33.3–143.0); *n* = 748	0.479
**CD19^+^CD20^+^CD138^+^** (/mL)	819.0 (334.0–1811.5); *n* = 749	1127.0 (137.3–2752.5); *n* = 8	806.0 (335.–1807.5); *n* = 741	0.974
**CD19^+^CD20^-^CD138^+^** (/mL)	482.5 (211.0–1067.3); *n* = 748	589.5 (242.3–1740.0); *n* = 8	480.5 (211.0–1058.8); *n* = 740	0.464
**CD19^+^CD20^-^CD27^brt^** (/mL)	299.0 (115.0–705.0); *n* = 747	365.0 (119.3–446.8); *n* = 8	298.0 (115.0–707.0); *n* = 739	0.838
**CD19^+^CD27^brt^CD38^brt^** (/mL)	306.0 (116.0–701.8); *n* = 754	326.5 (159.0–402.8); *n* = 8	306.0 (115.8–706.0); *n* = 746	0.804
**BLISS-SC**				
	*n* = **836**	*n* = **47**	*n* = **789**	
**CD19^+^CD20^+^** (×10^3^/mL)	106.0 (56.0–196.0); *n* = 811	91.0 (41.3–270.5); *n* = 44	107.0 (57.0–194.0); *n* = 767	0.680
**CD19^+^CD20^+^CD27^+^** (×10^3^/mL)	14.0 (7.0–29.0); *n* = 811	10.5 (5.3–28.8); *n* = 44	14.0 (7.0–29.0); *n* = 767	0.450
**CD19^+^CD20^+^CD69^+^** (/mL)	79.0 (33.0–199.0); *n* = 811	47.5 (23.5–137.3); *n* = 44	80.0 (34.0–202.0); *n* = 767	0.041
**CD19^+^CD20^+^CD27^-^** (×10^3^/mL)	89.0 (43.0–167.0); *n* = 811	81.0 (24.8–239.8); *n* = 44	90.0 (43.0–166.0); *n* = 767	0.819
**CD19^+^CD20^+^CD138^+^** (/mL)	53.0 (20.0–127.0); *n* = 811	63.0 (23.3–152.8); *n* = 44	53.0 (20.0–126.0); *n* = 767	0.479
**CD19^+^CD20^-^CD138^+^** (/mL)	203.0 (67.0–505.0); *n* = 811	253.5 (46.3–698.8); *n* = 44	201.0 (68.0–501.0); *n* = 767	0.496
**CD19^+^CD20^-^CD27^brt^** (/mL)	2000.0 (1000.0–4000.0); *n* = 811	2000.0 (1000.0–7000.0); *n* = 44	2000.0 (1000.0–4000.0); *n* = 767	0.060
**CD19^+^CD27^brt^CD38^brt^** (/mL)	1732.0 (738.0–3926.0); *n* = 811	2442.0 (738.5–7416.3); *n* = 44	1714.0 (731.0–3793.0); *n* = 767	0.053
**BLISS Northeast Asia**				
	*n* = **60**	*n* = **8**	*n* = **52**	
**CD19^+^CD20^+^** (×10^3^/mL)	52.5 (22.8–96.8); *n* = 54	65.0 (12.0–80.0); *n* = 5	51.0 (25.5–105.0); *n* = 49	0.467
**CD19^+^CD20^+^CD27^+^** (×10^3^/mL)	7.3 (3.7–10.6); *n* = 55	11.0 (3.3–19.1); *n* = 6	7.3 (3.5–10.5); *n* = 49	0.703
**CD19^+^CD20^+^CD69^+^** (/mL)	101.3 (45.9–183.0); *n* = 55	124.3 (77.0–170.2); *n* = 6	100.7 (45.6–185.4); *n* = 49	0.782
**CD19^+^CD20^+^CD27^-^** (×10^3^/mL)	39.7 (18.6–87.5); *n* = 55	23.5 (5.6–58.2); *n* = 6	41.4 (19.8–98.4); *n* = 49	0.104
**CD19^+^CD20^+^CD138^+^** (/mL)	108.2 (58.1–258.1); *n* = 55	167.8 (113.0–599.3); *n* = 6	86.9 (54.0–218.7); *n* = 49	0.077
**CD19^+^CD20^-^CD138^+^** (/mL)	303.1 (174.5–668.8); *n* = 55	269.5 (49.7–467.4); *n* = 6	303.1 (176.7–698.3); *n* = 49	0.375
**CD19^+^CD20^-^CD27^brt^** (/mL)	916.5 (262.8–2008.4); *n* = 55	1122.8 (315.0–1730.9); *n* = 6	904.3 (240.7–2446.1); *n* = 49	0.969
**CD19^+^CD27^brt^CD38^brt^** (/mL)	934.9 (264.7–2095.6); *n* = 55	1161.5 (132.1–1867.2); *n* = 6	887.6 (274.6–2177.5); *n* = 49	0.969

Data are presented as medians (interquartile range) of absolute counts. In case of missing values, the total number of patients with available data is indicated. *p* values are derived from non-parametrical Mann–Whitney U tests. Statistically significant *p* values are in bold. brt: bright; SC: subcutaneous.

## Data Availability

Data are available upon request through the Clinical Study Data Request (CSDR) portal: https://www.clinicalstudydatarequest.com (accessed on 13 August 2022).

## Supplementary Materials

The following supporting information can be downloaded at: https://www.mdpi.com/article/10.3390/ijms232213941/s1.
