# Supplementary material for: B Cell Kinetics upon Therapy Commencement for Active Extrarenal Systemic Lupus Erythematosus in Relation to Development of Renal Flares: Results from Three Phase III Clinical Trials of Belimumab
Source: Int J Mol Sci. 2022 Nov 11;23(22):13941. doi: 10.3390/ijms232213941 (PMC9698874; doi:10.3390/ijms232213941)
Supplement: Supplementary file 1 [file ijms-23-13941-s001.zip › Supplementary Table S1.pdf]

Supplementary Table S1. Disease activity across the organ domains of the classic BILAG index.

BILAG domain	BILAG index score				
	A	B	C	D	E
General	4 (0.2%)	175 (10.2%)	839 (48.9%)	37 (2.2%)	660 (38.5%)
Mucocutaneous	98 (5.7%)	981 (57.2%)	441 (25.7%)	24 (1.4%)	171 (10.0%)
Neurological	5 (0.3%)	20 (1.2%)	191 (11.1%)	22 (1.3%)	1477 (86.1%)
Musculoskeletal	122 (7.1%)	1084 (63.2%)	304 (17.7%)	27 (1.6%)	178 (10.4%)
Cardiovascular	7 (0.4%)	50 (2.9%)	210 (12.2%)	41 (2.4%)	1407 (82.0%)
Vascular	38 (2.2%)	114 (6.6%)	574 (33.5%)	46 (2.7%)	943 (55.0%)
Renal	10 (0.6%)	142 (8.3%)	383 (22.3%)	98 (5.7%)	1082 (63.1%)
Haematological	2 (0.1%)	254 (14.8%)	647 (37.7%)	100 (5.8%)	712 (41.5%)

Data are presented as numbers (percentage).

BILAG: British Isles Lupus Assessment Group.
