# Supplementary material for: B Cell Kinetics upon Therapy Commencement for Active Extrarenal Systemic Lupus Erythematosus in Relation to Development of Renal Flares: Results from Three Phase III Clinical Trials of Belimumab
Source: Int J Mol Sci. 2022 Nov 11;23(22):13941. doi: 10.3390/ijms232213941 (PMC9698874; doi:10.3390/ijms232213941)
Supplement: Supplementary file 1 [file ijms-23-13941-s001.zip › Supplementary Table S10.pdf]

Supplementary Table S10. Relative to baseline percentage changes through week 8, 24, and 52 in peripheral B cell subset counts and serum levels of serological markers in patients who developed renal flares versus patients who did not from baseline through week 52 in the BLISS-SC study population.

B cell subsets	Renal flare	No renal flare	P value
**Baseline through week 8**			
**Entire patient cohort** (all treatment arms)			
$CD19^{+}CD20^{+}CD27^{-}$	-25.0 (-45.2−26.9)	-14.9 (-44.6−16.9)	0.833
$CD19^{+}CD27^{-}CD24^{lo}CD38^{lo}$	-43.3 (-56.8−20.6)	-19.8 (-50.5−19.5)	0.226
$CD19^{+}CD27^{-}CD24^{brt}CD38^{brt}$	-34.5 (-72.3−50.0)	-57.9 (-87.5−0.0)	**0.046**
**Belimumab**			
$CD19^{+}CD20^{+}CD27^{-}$	-27.8 (-53.8−6.3)	-26.2 (-50.4−13.8)	0.803
$CD19^{+}CD27^{-}CD24^{lo}CD38^{lo}$	-45.3 (-55.7−-9.4)	-33.9 (59.2−10.0)	0.609
$CD19^{+}CD27^{-}CD24^{brt}CD38^{brt}$	-42.9 (-76.3−6.3)	-75.0 (-93.3−-33.3)	**0.038**
**Placebo**			
$CD19^{+}CD20^{+}CD27^{-}$	-7.1 (-35.3−59.0)	-2.7 (-22.3−29.9)	0.769
$CD19^{+}CD27^{-}CD24^{lo}CD38^{lo}$	-42.9 (-59.8−60.3)	0.0 (25.7−33.5)	0.172
$CD19^{+}CD27^{-}CD24^{brt}CD38^{brt}$	-7.1 (-69.8−137.5)	0.0 (-54.7−58.6)	0.871
**Belimumab vs placebo** (P value)			
$CD19^{+}CD20^{+}CD27^{-}$	0.231	**<0.001**	N/A
$CD19^{+}CD27^{-}CD24^{lo}CD38^{lo}$	0.587	**<0.001**	N/A
$CD19^{+}CD27^{-}CD24^{brt}CD38^{brt}$	0.146	**<0.001**	N/A
**Baseline through week 24**			
**Entire patient cohort** (all treatment arms)			
$CD19^{+}CD20^{+}CD27^{-}$	-20.0 (-59.5−26.0)	-41.4 (-66.3−-1.2)	0.193
$CD19^{+}CD27^{-}CD24^{lo}CD38^{lo}$	-43.0 (-72.7−18.0)	-50.0 (-73.5−-11.1)	0.335
$CD19^{+}CD27^{-}CD24^{brt}CD38^{brt}$	-57.4 (-87.7−0.0)	-62.5 (-92.2−0.0)	0.454
**Belimumab**			
$CD19^{+}CD20^{+}CD27^{-}$	-50.5 (-75.0−8.3)	-54.8 (-73.0−-29.4)	0.467
$CD19^{+}CD27^{-}CD24^{lo}CD38^{lo}$	-57.2 (-81.0−-37.1)	-63.6 (-79.6−-34.9)	0.617
$CD19^{+}CD27^{-}CD24^{brt}CD38^{brt}$	-69.0 (-96.3−0.0)	-75.0 (-100.0−-44.4)	0.562
**Placebo**			
$CD19^{+}CD20^{+}CD27^{-}$	1.6 (-42.7−36.1)	-5.5 (-38.5−29.8)	0.913
$CD19^{+}CD27^{-}CD24^{lo}CD38^{lo}$	12.1 (-50.0−152.1)	-10.1 (-40.7−43.0)	0.821
$CD19^{+}CD27^{-}CD24^{brt}CD38^{brt}$	-37.5 (-80.5−23.1)	-21.1 (-62.6−50.0)	0.647
**Belimumab vs placebo** (P value)			
$CD19^{+}CD20^{+}CD27^{-}$	0.074	**<0.001**	N/A
$CD19^{+}CD27^{-}CD24^{lo}CD38^{lo}$	**0.018**	**<0.001**	N/A
$CD19^{+}CD27^{-}CD24^{brt}CD38^{brt}$	0.193	**<0.001**	N/A
**Baseline through week 52**			
**Entire patient cohort** (all treatment arms)			
$CD19^{+}CD20^{+}CD27^{-}$	-33.9 (-73.7−2.0)	-58.9 (-79.0−-16.3)	0.368
$CD19^{+}CD27^{-}CD24^{lo}CD38^{lo}$	-40.7 (-87.7−0.0)	-67.6 (-85.7−-25.7)	0.386
$CD19^{+}CD27^{-}CD24^{brt}CD38^{brt}$	-50.4 (-85.7−0.0)	-55.8 (-90.7−0.0)	0.609
**Belimumab**			
$CD19^{+}CD20^{+}CD27^{-}$	-70.0 (-90.2−-19.1)	-70.4 (-83.4−-51.5)	0.881
$CD19^{+}CD27^{-}CD24^{lo}CD38^{lo}$	-77.4 (-93.7−-51.9)	-80.0 (-89.4−-61.9)	0.769
$CD19^{+}CD27^{-}CD24^{brt}CD38^{brt}$	-50.0 (-82.1−0.0)	-71.4 (-97.7−-25.0)	0.256
**Placebo**			
$CD19^{+}CD20^{+}CD27^{-}$	-7.1 (-47.7−59.6)	-11.4 (-42.3−37.4)	0.792
$CD19^{+}CD27^{-}CD24^{lo}CD38^{lo}$	0.0 (-27.7−35.4)	-18.2 (-52.3−44.4)	0.518
$CD19^{+}CD27^{-}CD24^{brt}CD38^{brt}$	-67.3 (-91.7−487.5)	0.0 (-52.8−100.0)	0.503
**Belimumab vs placebo** (P value)			
$CD19^{+}CD20^{+}CD27^{-}$	**0.018**	**<0.001**	N/A
$CD19^{+}CD27^{-}CD24^{lo}CD38^{lo}$	**0.007**	**<0.001**	N/A
$CD19^{+}CD27^{-}CD24^{brt}CD38^{brt}$	0.864	**<0.001**	N/A

Data are presented as medians (interquartile range) of the relative to baseline percentage changes. P values are derived from non-parametrical Mann-Whitney *U* tests. Statistically significant P values are in bold.

C3: complement component 3; C4: complement component 4; N/A: not applicable, SC: subcutaneous.
