# Supplementary material for: B Cell Kinetics upon Therapy Commencement for Active Extrarenal Systemic Lupus Erythematosus in Relation to Development of Renal Flares: Results from Three Phase III Clinical Trials of Belimumab
Source: Int J Mol Sci. 2022 Nov 11;23(22):13941. doi: 10.3390/ijms232213941 (PMC9698874; doi:10.3390/ijms232213941)
Supplement: Supplementary file 1 [file ijms-23-13941-s001.zip › Supplementary Table S2.pdf]

Supplementary Table S2. Characteristics of patients who developed renal flares versus patients who did not from baseline through week 76 in the pooled BLISS study population.

	All patients N=1715	Renal flare N=69	No renal flare N=1646	P value
**Patient characteristics**				
Age at baseline (years)	39.3 ± 11.9	34.9 ± 12.0	39.5 ± 11.8	**0.001**
Female sex	1608 (93.8%)	67 (97.1%)	1541 (93.6%)	0.316
Ancestry				
Asian	270 (15.7%)	28 (40.6%)	242 (14.7%)	**<0.001**
Black/African American	204 (11.9%)	8 (11.6%)	196 (11.9%)	0.937
Indigenous American*	170 (9.9%)	7 (10.1%)	163 (9.9%)	0.947
White/Caucasian	1071 (62.4%)	26 (37.7%)	1045 (63.5%)	**0.000**
**Clinical data**				
SLE duration at baseline (years)	5.1 (1.6−10.6)	3.5 (1.0−9.6)	5.1 (1.6−10.6)	0.098
BILAG renal				
A	10 (0.6%)	2 (2.9%)	8 (0.5%)	0.058
B	142 (8.3%)	16 (23.2%)	126 (7.6%)	**<0.001**
C	383 (22.3%)	30 (43.5%)	353 (21.4%)	**<0.001**
D	98 (5.7%)	8 (11.6%)	90 (5.5%)	**0.032**
E	1082 (63.1%)	13 (18.8%)	1069 (64.9%)	**<0.001**
A–B	152 (8.9%)	18 (28.1%)	134 (8.1%)	**<0.001**
Treatment at baseline				
Glucocorticoids or equivalent	1405 (81.9%)	63 (91.3%)	1342 (81.5%)	**0.039**
AMA†	1099 (64.1%)	38 (55.1%)	1061 (64.5%)	0.111
Immunosuppressants‡	882 (51.4%)	43 (62.3%)	839 (51.0%)	0.065
Azathioprine	336 (19.6%)	16 (23.2%)	320 (19.4%)	0.442
Methotrexate	248 (14.5%)	8 (11.6%)	240 (14.6%)	0.490
Mycophenolate mofetil or sodium	243 (14.2%)	14 (20.3%)	229 (13.9%)	0.137
Trial intervention				
Placebo	576 (33.6%)	30 (43.5%)	546 (33.2%)	0.076
Belimumab	1139 (66.4%)	39 (56.5%)	1100 (66.8%)	0.076
i.v. 1 mg/kg (every 4th week)	271 (15.8%)	2 (2.9%)	269 (16.3%)	**0.003**
i.v. 10 mg/kg (every 4th week)	312 (18.2%)	11 (15.9%)	301 (18.3%)	0.621
s.c. 200 mg (weekly)	556 (32.4%)	26 (37.7%)	530 (32.2%)	0.341
**Serological markers at baseline**				
C3; mg/dL	95.0 (74.0−118.0)	75.0 (57.5−95.5)	96.0 (75.0−119.0)	**<0.001**
C4; mg/dL	15.0 (9.0−22.0)	11.0 (7.0−16.5)	15.0 (9.0−22.0)	**<0.001**
anti-dsDNA; IU/mL (all patients)	95.0 (29.0−288.0)	236.0 (88.5−600.0)	90.0 (29.0−275.3)	**<0.001**
anti-dsDNA; IU/mL (patients positive at baseline)	167.0 (89.0−497.3); N=1172	279.0 (136.0−663.5); N=58	163.5 (87.5−491.5); N=1114	**0.005**

Data are presented as the number (percentage), mean ± standard deviation, or median (interquartile range), as appropriate. In case of missing values, the total number of patients with available data is indicated. Percentages are derived using the total number of patients in the respective column as the denominator (i.e., all patients, patients who developed renal flares, and patients who did not develop renal flares). In case of missing values, the total number of patients with available data is indicated. Statistically significant P values are in bold.

* Alaska Native or American Indian from North, South or Central America.
† Hydroxychloroquine, chloroquine, mepacrine, mepacrine hydrochloride or quinine sulfate.
‡ Azathioprine, cyclosporine, oral cyclophosphamide, leflunomide, methotrexate, mizoribine, mycophenolate mofetil, mycophenolate sodium or thalidomide.

AMA: antimalarial agents; C3: complement component 3; C4: complement component 4; i.v.: intravenous; s.c.: subcutaneous; SLE: systemic lupus erythematosus; SRI-4: SLE Responder Index 4.
