# Supplementary material for: B Cell Kinetics upon Therapy Commencement for Active Extrarenal Systemic Lupus Erythematosus in Relation to Development of Renal Flares: Results from Three Phase III Clinical Trials of Belimumab
Source: Int J Mol Sci. 2022 Nov 11;23(22):13941. doi: 10.3390/ijms232213941 (PMC9698874; doi:10.3390/ijms232213941)
Supplement: Supplementary file 1 [file ijms-23-13941-s001.zip › Supplementary Table S3.pdf]

Supplementary Table S3. B cell subset counts at baseline in patients who developed renal flares versus patients who did not from baseline through week 76 in the BLISS-76, BLISS-SC, and BLISS Northeast Asia study populations.

B cell subsets	All patients	Renal flare	No renal flare	P value
		**BLISS-76**		
	**N=819**	**N=14**	**N=805**	
**CD19$^+$CD20$^+$** (x10^3/mL)	91.5 (43.0−176.0); N=756	96.0 (37.8−164.3); N=12	91.0 (43.3−177.5); N=744	0.881
**CD19$^+$CD20$^+$CD27$^+$** (x10^3/mL)	14.0 (6.0−27.0); N=756	17.0 (7.8−25.5); N=12	14.0 (6.0−27.0); N=744	0.822
**CD19$^+$CD20$^+$CD69$^+$** (/mL)	2096.5 (938.3−4350.8); N=744	2424.5 (708.3−9099.3); N=12	2094.5 (938.3−4327.0); N=732	0.468
**CD19$^+$CD20$^+$CD27$^-$** (x10^3/mL)	75.0 (33.0−143.0); N=756	77.5 (26.3−142.3); N=12	75.0 (33.3−143.0); N=744	0.881
**CD19$^+$CD20$^+$CD138$^+$** (/mL)	819.0 (334.0−1811.5); N=749	1196.5 (265.0−2203.5); N=12	805.0 (334.0−1807.5); N=737	0.676
**CD19$^+$CD20$^-$CD138$^+$** (/mL)	482.5 (211.0−1067.3); N=748	589.5 (321.0−1667.3); N=12	478.0 (208.8−1058.8); N=736	0.319
**CD19$^+$CD20$^-$CD27^{brt}** (/mL)	299.0 (115.0−705.0); N=747	365.0 (208.0−470.5); N=12	296.0 (115.0−707.0); N=735	0.663
**CD19$^+$CD27^{brt}CD38^{brt}** (/mL)	306.0 (116.0−701.8); N=754	375.5 (208.0−506.0); N=12	305.0 (115.0−706.0); N=742	0.627
		**BLISS-SC**		
	**N=836**	**N=47**	**N=789**	
**CD19$^+$CD20$^+$** (x10^3/mL)	106.0 (56.0−196.0); N=811	91.0 (41.3−270.5); N=44	107.0 (57.0−194.0); N=767	0.680
**CD19$^+$CD20$^+$CD27$^+$** (x10^3/mL)	14.0 (7.0−29.0); N=811	10.5 (5.3−28.8); N=44	14.0 (7.0−29.0); N=767	0.450
**CD19$^+$CD20$^+$CD69$^+$** (/mL)	79.0 (33.0−199.0); N=811	47.5 (23.5−137.3); N=44	80.0 (34.0−202.0); N=767	**0.041**
**CD19$^+$CD20$^+$CD27$^-$** (x10^3/mL)	89.0 (43.0−167.0); N=811	81.0 (24.8−239.8); N=44	90.0 (43.0−166.0); N=767	0.819
**CD19$^+$CD20$^+$CD138$^+$** (/mL)	53.0 (20.0−127.0); N=811	63.0 (23.3−152.8); N=44	53.0 (20.0−126.0); N=767	0.479
**CD19$^+$CD20$^-$CD138$^+$** (/mL)	203.0 (67.0−505.0); N=811	253.5 (46.3−698.8); N=44	201.0 (68.0−501.0); N=767	0.496
**CD19$^+$CD20$^-$CD27^{brt}** (/mL)	2000.0 (1000.0−4000.0); N=811	2000.0 (1000.0−7000.0); N=44	2000.0 (1000.0−4000.0); N=767	0.060
**CD19$^+$CD27^{brt}CD38^{brt}** (/mL)	1732.0 (738.0−3926.0); N=811	2442.0 (738.5−7416.3); N=44	1714.0 (731.0−3793.0); N=767	0.053
		**BLISS Northeast Asia**		
	**N=60**	**N=8**	**N=52**	
**CD19$^+$CD20$^+$** (x10^3/mL)	52.5 (22.8−96.8); N=54	65.0 (12.0−80.0); N=5	51.0 (25.5−105.0); N=49	0.467
**CD19$^+$CD20$^+$CD27$^+$** (x10^3/mL)	7.3 (3.7−10.6); N=55	11.0 (3.3−19.1); N=6	7.3 (3.5−10.5); N=49	0.703
**CD19$^+$CD20$^+$CD69$^+$** (/mL)	101.3 (45.9−183.0); N=55	124.3 (77.0−170.2); N=6	100.7 (45.6−185.4); N=49	0.782
**CD19$^+$CD20$^+$CD27$^-$** (x10^3/mL)	39.7 (18.6−87.5); N=55	23.5 (5.6−58.2); N=6	41.4 (19.8−98.4); N=49	0.104
**CD19$^+$CD20$^+$CD138$^+$** (/mL)	108.2 (58.1−258.1); N=55	167.8 (113.0−599.3); N=6	86.9 (54.0−218.7); N=49	0.077
**CD19$^+$CD20$^-$CD138$^+$** (/mL)	303.1 (174.5−668.8); N=55	269.5 (49.7−467.4); N=6	303.1 (176.7−698.3); N=49	0.375
**CD19$^+$CD20$^-$CD27^{brt}** (/mL)	916.5 (262.8−2008.4); N=55	1122.8 (315.0−1730.9); N=6	904.3 (240.7−2446.1); N=49	0.969
**CD19$^+$CD27^{brt}CD38^{brt}** (/mL)	934.9 (264.7−2095.6); N=55	1161.5 (132.1−1867.2); N=6	887.6 (274.6−2177.5); N=49	0.969

Data are presented as medians (interquartile range) of absolute counts. In case of missing values, the total number of patients with available data is indicated. P values are derived from non-parametrical Mann-Whitney *U* tests. Statistically significant P values are in bold.

SC: subcutaneous.
