# Supplementary material for: B Cell Kinetics upon Therapy Commencement for Active Extrarenal Systemic Lupus Erythematosus in Relation to Development of Renal Flares: Results from Three Phase III Clinical Trials of Belimumab
Source: Int J Mol Sci. 2022 Nov 11;23(22):13941. doi: 10.3390/ijms232213941 (PMC9698874; doi:10.3390/ijms232213941)
Supplement: Supplementary file 1 [file ijms-23-13941-s001.zip › Supplementary Table S9.pdf]

Supplementary Table S9. Relative to baseline percentage changes through week 52 in B cell subset counts and serum levels of serological markers in patients who developed renal flares versus patients who did not through week 76 in the pooled BLISS study population.

	Renal flare	No renal flare	Coefficient	OR	95% CI	P value
**Entire patient cohort** (all treatment arms)						
**B cell subsets**						
**CD19^{+}CD20^{+}**	-24.0 (-74.6–44.1)	-40.0 (-66.0–-1.0)	0.06	1.06	0.60–1.85	0.844
**CD19^{+}CD20^{+}CD27^{+}**	24.8 (-44.4–177.5)	15.4 (-28.0–90.9)	0.13	1.14	0.89–1.47	0.312
**CD19^{+}CD20^{+}CD69^{+}**	5.5 (-65.7–97.0)	-41.3 (-79.4–42.4)	0.04	1.04	0.92–1.17	0.522
**CD19^{+}CD20^{+}CD27^{-}**	-52.1 (-76.6–-7.7)	-56.1 (-77.7–-14.3)	-0.12	0.89	0.43–1.84	0.756
**CD19^{+}CD20^{+}CD138^{+}**	-24.7 (-80.3–124.6)	-50.0 (-81.8–27.9)	0.05	1.05	0.93–1.20	0.427
**CD19^{+}CD20^{-}CD138^{+}**	-54.3 (-85.9–22.0)	-44.9 (-77.9–42.2)	0.01	1.01	0.85–1.21	0.902
**CD19^{+}CD20^{-}CD27^{brt}**	-50.0 (-79.5–-7.1)	-38.0 (-77.3–16.0)	-0.23	0.80	0.56–1.14	0.216
**CD19^{+}CD27^{brt}CD38^{brt}**	-42.7 (-77.9–4.5)	-34.5 (-71.2–40.1)	-0.20	0.82	0.61–1.11	0.201
**Serological markers**						
**C3**	20.9 (-4.2–51.9)	4.1 (-7.7–17.9)	1.56	4.75	1.97–11.5	**0.001**
**C4**	42.3 (-6.8–95.0)	14.3 (-3.4–40.0)	0.46	1.58	1.11–2.23	**0.010**
**anti-dsDNA** (all patients)	-37.5 (-61.8–21.7)	-22.4 (-55.0–0.0)	0.03	1.03	0.96–1.09	0.424
**anti-dsDNA** (patients positive at baseline)	-43.6 (-61.8–21.7)	-42.0 (-65.2–-10.7)	0.06	1.06	0.95–1.18	0.307
**Belimumab**						
**B cell subsets**						
**CD19^{+}CD20^{+}**	-47.1 (-78.4–34.8)	-52.9 (-71.4–-19.1)	0.21	1.24	0.61–2.52	0.555
**CD19^{+}CD20^{+}CD27^{+}**	70.2 (-30.4–225.2)	32.9 (-14.3–114.5)	0.17	1.19	0.89–1.58	0.239
**CD19^{+}CD20^{+}CD69^{+}**	2.8 (-80.8–93.1)	-44.2 (-81.3–38.9)	0.02	1.02	0.86–1.22	0.801
**CD19^{+}CD20^{+}CD27^{-}**	-68.8 (-87.5–-44.0)	-69.2 (-82.3–-48.1)	-0.10	0.91	0.27–3.00	0.874
**CD19^{+}CD20^{+}CD138^{+}**	-44.9 (-89.5–49.1)	-56.3 (-83.2–8.3)	0.03	1.03	0.86–1.24	0.735
**CD19^{+}CD20^{-}CD138^{+}**	-71.9 (-91.6–10.4)	-48.6 (-80.1–22.4)	0.09	1.10	0.89–1.36	0.391
**CD19^{+}CD20^{-}CD27^{brt}**	-58.8 (-88.1–-29.4)	-50.0 (-83.3–0.7)	-0.47	0.62	0.29–1.32	0.217
**CD19^{+}CD27^{brt}CD38^{brt}**	-55.2 (-87.0–-20.3)	-45.8 (-75.9–23.4)	-0.38	0.68	0.37–1.25	0.216
**Serological markers**						
**C3**	25.0 (-1.1–46.2)	6.4 (-5.1–22.1)	1.65	5.19	1.77–15.25	**0.003**
**C4**	55.6 (16.7–100.0)	20.0 (0.0–46.7)	0.63	1.88	1.13–3.13	**0.015**
**anti-dsDNA** (all patients)	-51.1 (-67.9–-4.3)	-30.8 (-61.7–0.0)	0.01	1.01	0.92–1.11	0.835
**anti-dsDNA** (patients positive at baseline)	-50.7 (-70.3–-5.1)	-47.6 (-67.4–-20.7)	0.03	1.03	0.82–1.29	0.790
**Placebo**						
**B cell subsets**						
**CD19^{+}CD20^{+}**	-8.5 (-58.3–56.8)	-10.5 (-40.0–31.4)	-0.18	0.84	0.35–2.02	0.696
**CD19^{+}CD20^{+}CD27^{+}**	-27.3 (-48.7–100.0)	-12.5 (-44.8–50.0)	0.01	1.01	0.59–1.72	0.977
**CD19^{+}CD20^{+}CD69^{+}**	15.8 (-10.0–124.2)	-34.4 (-74.0–57.2)	0.06	1.06	0.90–1.26	0.490
**CD19^{+}CD20^{+}CD27^{-}**	-10.0 (-61.5–72.2)	-10.5 (-40.1–31.5)	-0.14	0.87	0.35–2.18	0.766
**CD19^{+}CD20^{+}CD138^{+}**	57.8 (-49.5–200.6)	-28.4 (-78.2–73.4)	0.07	1.08	0.90–1.29	0.435
**CD19^{+}CD20^{-}CD138^{+}**	-43.3 (-74.7–25.8)	-30.2 (-69.6–81.5)	-0.11	0.90	0.65–1.24	0.512
**CD19^{+}CD20^{-}CD27^{brt}**	-50.0 (-75.0–44.4)	-15.6 (-54.8–65.2)	-0.12	0.89	0.61–1.31	0.555
**CD19^{+}CD27^{brt}CD38^{brt}**	-24.9 (-77.6–74.7)	-11.1 (-55.3–90.1)	-0.10	0.90	0.64–1.27	0.561
**Serological markers**						
**C3**	10.3 (-34.0–55.3)	-1.5 (-11.4–10.7)	1.26	3.52	0.73–17.0	0.117
**C4**	10.5 (-37.4–73.2)	0.0 (-14.3–20.4)	0.32	1.37	0.79–2.40	0.266
**anti-dsDNA** (all patients)	-9.3 (-58.3–32.5)	0.0 (-41.3–5.2)	0.21	1.23	1.00–1.51	**0.045**
**anti-dsDNA** (patients positive at baseline)	-18.6 (-59.6–31.7)	-22.8 (-50.0–11.3)	0.24	1.27	0.93–1.72	0.133
**Belimumab vs placebo** (P value)						
**B cell subsets**						
**CD19^{+}CD20^{+}**	0.202	**<0.001**	N/A	N/A	N/A	N/A
**CD19^{+}CD20^{+}CD27^{+}**	0.107	**<0.001**	N/A	N/A	N/A	N/A
**CD19^{+}CD20^{+}CD69^{+}**	0.364	**0.030**	N/A	N/A	N/A	N/A
**CD19^{+}CD20^{+}CD27^{-}**	**0.004**	**<0.001**	N/A	N/A	N/A	N/A
**CD19^{+}CD20^{+}CD138^{+}**	0.180	**<0.001**	N/A	N/A	N/A	N/A
**CD19^{+}CD20^{-}CD138^{+}**	0.319	**<0.001**	N/A	N/A	N/A	N/A
**CD19^{+}CD20^{-}CD27^{brt}**	0.166	**<0.001**	N/A	N/A	N/A	N/A
**CD19^{+}CD27^{brt}CD38^{brt}**	0.139	**<0.001**	N/A	N/A	N/A	N/A
**Serological markers**						
**C3**	0.254	**<0.001**	N/A	N/A	N/A	N/A
**C4**	**0.030**	**<0.001**	N/A	N/A	N/A	N/A
**anti-dsDNA** (all patients)	0.106	**<0.001**	N/A	N/A	N/A	N/A
**anti-dsDNA** (patients positive at baseline)	0.274	**<0.001**	N/A	N/A	N/A	N/A

Results from multivariable logistic regression analysis with renal flare from baseline through week 76 as the dependent variable. Apart from the main exposure under investigation i.e., relative to baseline percentage changes through week 52 in B cell subset counts or serum levels of serological markers, other covariates in the models included age, ethnicity, glucocorticoid use, and belimumab use; adjustment for belimumab use was not applicable in models stratified by treatment arm. Relative to baseline percentage changes from baseline through week 52 in B cell subset counts and serum levels of serological markers are presented as medians (interquartile range). Results from the logistic regression analysis are presented as the coefficient, odds ratio (OR), 95% confidence interval (CI) and P value for the main exposure in the respective multivariable logistic regression model. Comparisons between treatment arms were conducted using non-parametrical Mann-Whitney *U* tests. Statistically significant P values are in bold. C3: complement component 3; C4: complement component 4; CI: confidence interval; N/A: not applicable; OR: odds ratio.
